# Effects of Blast Furnace Slag Powder and Limestone Powder on the Mechanical Properties and Durability of Shotcrete Using Monocalcium Aluminate Setting Accelerator

**DOI:** 10.3390/ma15072495

**Published:** 2022-03-28

**Authors:** Bonghee Kang, Gyuyong Kim, Taegyu Lee, Kyungmo Koo, Sangkyu Lee, Minjae Son, Sasui Sasui, Hamin Eu

**Affiliations:** 1 Asia Cement Co., Ltd., 14 Songhaksan-ro, Songhak-myeon, Jecheon-si 27125, Korea; kangbh@asiacement.co.kr; 2 Department of Architectural Engineering, Chungnam National University, 99 Daehak-ro, Yuseong-gu, Daejeon 34134, Korea; minjae931226@naver.com (M.S.); sassuikhuwaja126@gmail.com (S.S.); wp05125@naver.com (H.E.); 3 Department of Fire and Disaster Prevention, Semyung University, 65 Semyung-ro, Jecheon-si 27136, Korea; ltg777@semyung.ac.kr; 4 Building Engineering Team, Hanwha Engineering and Construction, 86 Cheonggyecheon-ro, Seoul 04541, Korea; kkm@hanwha.com; 5 Korea Construction Standards Center, Korea Institute of Civil Engineering and Building Technology, 283, Goyang-daero, Ilsanseo-gu, Goyang-si 10223, Korea; sangkyulee@kict.re.kr

**Keywords:** shotcrete, calcium aluminate accelerator, ground granulated blast-furnace slag, limestone powder, cement hydration, durability

## Abstract

In this study, the effect of fine blast furnace slag powder (SP) and limestone powder (LSP) as a mineral admixture in shotcrete using monocalcium aluminate (CA) as a quick-setting accelerator was evaluated. The shotcrete was prepared with up to 25 wt.% substitutions of mineral admixture, i.e., (SP and LSP), and then the CA accelerator was incorporated by 5 wt.% of binders. To examine the optimal mixing ratio for mineral admixture in shotcrete, penetration resistance, compressive strength, XRD analysis, and MIP analysis were performed on the mortar. On the other hand, compressive strength test, chloride diffusion coefficient, and freeze–thaw resistance were conducted on concrete to evaluate the field applicability of shotcrete. The study revealed the addition of LSP improved setting time and early compressive strength while the addition of SP increased long-term compressive strength. With the addition of both SP and LSP, the early and long-term strength was increased due to the influence of the properties of each admixture. Furthermore, the addition of SP and LSP improves the resistance of shotcrete to chloride ions and freeze–thaw.

## 1. Introduction

Shotcrete or spray concrete is conveyed by spraying mortar or concrete onto the construction surface at high velocity. This technique is widely used for the stabilization of inclined surfaces and tunnel construction. Therefore, shotcrete requires rapid setting and rapid strength development [1,2]. In order to improve the early strength of shotcrete, it is essential to use an accelerator to minimize dust and rebound [3,4,5]. The agents used as an accelerator are formulated with an alkaline system, alkali-free system, and alkali silicate system. However, the long-term strength and durability performance of shotcrete can be affected by the presence of excessive alkali content in the system; therefore, the use of alkali-free accelerators is often preferred [6]. The non-alkaline accelerators consisting of calcium sulfo-aluminate or calcium aluminate are mainly used in dosage of up to 12% [7]. Calcium aluminate accelerators are mainly composed of C_12_A_7_ (Mayenite), monocalcium aluminate (CA), and grossite (CA2), which are minerals whose main components are Ca and Al. Among these accelerators, CA has a significant influence on the cement strength, and the hydration reaction proceeds step by step, as shown in Equation (1) [6].

(1) $$\mathrm{CA}\;\to {\mathrm{CAH}}_{10}\;\to {\;C}_{2}{\mathrm{AH}}_{8}+{\mathrm{AH}}_{3}\;\to {\;C}_{3}{\mathrm{AH}}_{6}+{\mathrm{AH}}_{3}$$

The use of an accelerator for rapid setting may ensure the stability of the construction unit through rapid strength development; however, in the case that there is a problem in adjusting the quick setting time, it may cause blockage of the pressure pipe and equipment failure. Therefore, the method to control the setting time is required. Generally, the calcium sulfate dihydrate (CaSO_4_·2H_2_O) or anhydrous gypsum (CaSO_4_) is added to control the setting time of Portland cement (OPC). This is because CAH_10_, a hydrated product of CA, reacts in the presence of calcium hydroxide (CH), an OPC hydrated product, as shown in Equation (2) [7].

(2) $${\mathrm{CAH}}_{10}+2\mathrm{CH}+3{\mathrm{Ca}\cdot \mathrm{SO}}_{4}+14H\;\to C_{3}A\cdot 3{\mathrm{CaSO}}_{4}\cdot H_{32}$$

In case when CH and CaSO_4_ are not supplied, the CAH_10_ will react to form C_2_AH_8_ and C_3_AH_6_, thereby reducing the strength. However, the supply of CH and CaSO_4_ from OPC in a certain range contributes to the formation of stable ettringite and improves the early strength. It also has been reported that CH and CaSO_4_ themselves promote the hydration reaction of CA [8]. Based on such a hydration reaction process, shotcrete using a CA accelerator is used in many tunnels.

Recently, shotcrete has been applied to areas directly exposed to seawater, such as undersea tunnels, and the durability of shotcrete is emerging to ensure the safety of structures [9]. Studies have been conducted on the use of mineral admixtures such as blast furnace slag (SP) and fly ash for shotcrete. Especially, SP has a potential hydraulic property and is known to contribute to the improvement of the long-term strength and durability of cement-based composite materials. On the other hand, incorporation of SP as a mineral admixture in shotcrete can affect the strength, and that may occur during the transition from CAH_10_ hydrate to C_3_AH_6_ by CA [10].

For the formation of C_3_AH_6_ from CAH_10_, Ca ions must be absorbed from the surroundings. However, with the hydration reaction of SP, the gehlenite hydrate, or C_2_ASH_8_ in the form of stratlingite develops, which interrupts the formation of C_3_AH_6_. In order to examine this mechanism, various studies have been conducted in which SP is incorporated into CAC. Edmonds et al. [11] studied the mixture of CA and SP and showed that the addition of SP C3AH6 was not produced at all after 60 days. As a result of conducting a transition reaction promotion test to C3AH6, Gosselin [12] reported that the addition of SP did not completely prevent the production of C3AH6 but effectively inhibited the production of C3AH6 as C2AH8 was produced.

Owing to the lower specific gravity of limestone powder (LSP), the volume of the paste and the distance between aggregate particles increase. Therefore, if an optimal proportion of LSP is incorporated in concrete, the workability of concrete can be improved, and W/B required to obtain the same workability can be reduced [13]. Schmidt et al. [14] showed improved workability of concrete when LSP content was below 17%. In the study by Moir et al. [15], the concrete obtained good workability when LSP content was below 20%. Furthermore, it has been reported that using LSP, which has higher fineness than OPC, can improve clinker reactivity, and can reduce the initial hydration reactivity caused by the nucleation effect [13].

Cussino et al. [16] conducted a study to explore the effects of LSP on the properties of concrete incorporating a CA accelerator. The study revealed that when LSP was added to OPC, monocarbonate (C_4_ACH_11_), which is a carbonate hydrate, was produced, which interrupted the transition of CAH_10_ to C_3_AH_6_. A similar phenomenon is shown in the study by Fentiman et al. [17], who discovered that the monocarbonate phase could have a positive effect on long-term durability.

Meanwhile, shotcrete is a rapid-set material having a reaction mechanism similar to concrete. In addition, it is expected to improve workability and economic efficiency by using admixtures such as SP and LSP. In addition, applicability may be increased by examining the non-hardened properties, compressive strength, durability of the shotcrete, but related studies are insignificant. In particular, LSP does not only works as a filler but also improves reactivity with CA, as reported in referenced studies [18,19]. Therefore, it is judged that it is necessary to study the effect.

Therefore, this study attempted to examine the effect of SP and LSP on the hydration reaction and mechanical properties of shotcrete using a CA setting accelerator. First, the effect on the hydration reaction was analyzed through the micro-structure analysis of the shotcrete binder (Mortar) according to the mixing rate of SP and LSP. After that, the effect of SP and LSP on durability was evaluated by examining the compressive strength, chloride diffusion coefficient, and freeze–thawing of shotcrete (concrete) containing CA as an accelerator, and, finally, the field applicability of shotcrete was examined.

## 2. Materials and Methods

### 2.1. Materials

In this study, Type 1 Portland cement (OPC, density: 3140 kg/m^3^, fineness: 348 m^2^/kg) was used as cement, ground granulated blast-furnace slag (SP, density: 2900 kg/m^3^, fineness: 414 m^2^/kg), and Limestone powder (LSP, density: 2800 kg/m^3^, fineness: 745 m^2^/kg) were used as a mineral admixture. Powder-type products (SC, density: 3010 kg/m^3^, and fineness: 502 m^2^/kg) containing CA, which is an alkali-free based material, were used as an accelerator. The chemical composition of these materials is shown in Table 1.

ISO Standard sand (density: 2620 kg/m^3^, SiO_2_: 99%, 0.08 mm passage amounts: 0.04%) was used as a fine aggregate to synthesize mortar [20]. While crushed sand (FM: 3.03, density: 2560 kg/m^3^, absorption: 0.64%) and crushed granular aggregate (size: 10 mm, density: 2650 kg/m^3^, absorption: 1.36%) was used to prepare concrete. To ensure working performance of concrete, the superplasticizer polycarboxylic acid group (density: 1218 kg/m^3^, solid content: 43.2%) was used.

### 2.2. Experimental Plan and Mix Proportion

The effect of CA accelerator on the hydration reaction of shotcrete in which the cement is replaced with a mineral admixture and the effects on the properties of a mortar and concrete for shotcrete was analyzed. As shown in Table 2, the test specimens prepared in series I are mortar specimens. In order to examine the effect of each mineral admixture, the SP 10% (SP10), 20% (SP20), and LSP with 5% (LSP5) and 10% (LSP10) was substituted with OPC by mass. The amount of CA accelerator for all specimens was fixed at 5% of the amount of binder. Hydrate analysis and pore structure analysis were performed through X-ray diffraction (XRD) analysis and Mercury intrusion porosimetry (MIP), respectively, and the mechanical properties of the specimen were evaluated through penetration resistance and compressive strength.

In order to examine the applicability of concrete, test specimen in Series II was set up by substituting OPC with 25% SP (SP25) and with 20% SP and 5% LSP (SP20LSP5). Similar to mortar, the amount of CA accelerator for all specimens was fixed at 5% of the amount of binder in concrete. The compressive strength, chloride ion penetration test, and freeze–thawing tests were conducted on concrete.

Table 3 shows the mix proportion for mortar. In order to prepare the mortar specimens, the dry-mix composed of 1350 g of sand and 450 g of binder was blended, then 225 g of water was added and mixed thoroughly for 5 min at approximately 21 °C. After 5 min of mixing, 22.5 g (5%, B × %) of accelerator was added and mixed the paste for 30 s.

Table 4 shows the mix proportion of concrete for shotcrete. Mixing of shotcrete was performed in accordance to ASTM C1436-13 [21], and some of the mix proportion was slightly changed by pre-experiments. To prepare concrete, the W/B = 0.43 and S/a = 60% was used. The concrete was prepared by dry-mixing binders, fine aggregate, and coarse aggregate, then pouring water and sufficiently mixing for 5 min, then adding an accelerator and re-mixing for 30 s at approximately 20 °C.

## 3. Results and Discussion

### 3.1. Properties of the Mortar

#### 3.1.1. Test Methods of Mortar Experiment

XRD was performed according to ASTM C1365 as a method for analyzing the hydration product of mortar for shotcrete [22]. Each sample was subjected to air curing for 1, 8 h and 1, 7, 28 days after the addition of a quick setting accelerator agent at 21 ± 1 °C and 85 ± 10% R.H. Then samples were immersed in acetone for 24 h to stop the hydration reaction. Finally, each sample was dried at 40 °C for 48 h. Analysis conditions were set at 2.4°/min range 5–65° under 40 kV and 250 mA and quantitatively analyzed using the Rietveld method. The mineral volume was quantitatively analyzed using thermogravimetric analysis (TGA) and differential thermal analysis (DTA). The pore structure of the shotcrete mortar was analyzed by using Mercury intrusion porosimetry (MIP) according to ASTM D4404 [23]. The setting time of Mortar was performed in accordance with ASTMC C403/C403M, and the penetration resistance was measured at 1, 3, and 5 min after the addition of the quick setting accelerator agent. The compressive strength of mortar was measured in accordance with ASTM C109/C109M using a universal test machine (UTM) [24,25].

#### 3.1.2. Analysis Result of Hydrate and Pore Distribution

**Figure 1:**
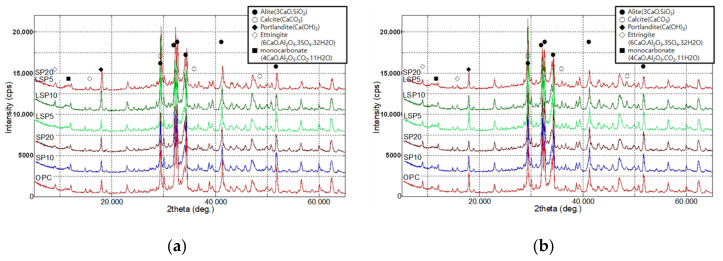
Compare of XRD pattern of mortar specimens at age: (**a**) 1 day; (**b**) 28 days.

XRD patterns of mortars after 1 day of curing and 28 days of curing are shown in Figure 1. The hydration reaction of cement affects the overall performance of the material, such as setting time and compressive strength, and the predominant chemical reaction at each age can be inferred by analyzing the reaction product produced by the hydration reaction. In all specimens, the peaks of Alite and Calcite, ettringite (Aft, (CaO)_6_(Al_2_O_3_)(SO_3_)_3_∙32H_2_O) monocarbonate (AFm, C3A·CaCO_3_·11H_2_O) and Portlandite (CH, Ca(OH)_2_) as crystalline hydrates were observed. In addition, the traces of hemicarbonate (C3A·0.5CaCO_3_·12H_2_O) and monosulfate (Ca_4_Al_2_SO_4_(OH)_12_) were also observed.

**Figure 2:**
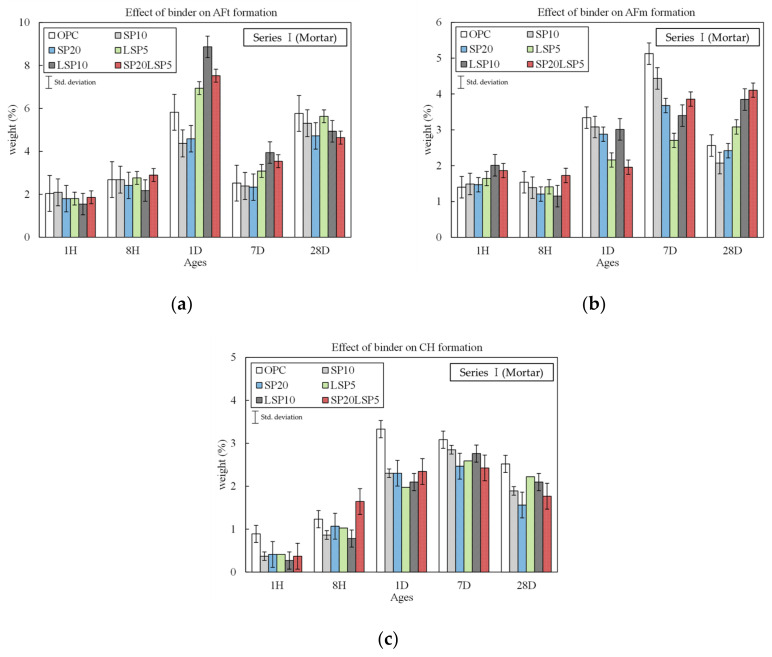
Amount of hydrate phases in each binder type: (**a**) Aft formation; (**b**) AFm formation; (**c**) CH formation.

Figure 2 shows the number of hydration phases in each binder type. Among the analyzed hydrates, the amount of AFt, AFm, and CH phases produced, with a clear difference, was selected as the basis.

In the case of mortar mixed with SP, there was no significant difference in weight of AFt and AFm products compared to OPC at 1, 8H ages, but CH products tended to be lower at 1, 8H. In 1 day of curing, it was confirmed that the AFt products were about 1.3% lower in both SP20 and 10. In the case of AFm products, it was confirmed that SP10 was 0.7% and SP20 was 1.5% lower. However, it was confirmed that there was no significant difference in the amount of AFt and AFm products in 28 days of curing. On the other hand, it was confirmed SP10, SP20 specimens tended to have a smaller amount of CH products than OPC specimens regardless of age. In particular, there was the biggest difference in the 1-day curing. This phenomenon is because the initial reactivity of the binder decreases due to the incorporation of SP, which has a slow reactivity, along with a decrease in the amount of OPC incorporated with the addition of SP. As a result, the production of AFt and AFm, which are the main hydrates in the initial stage of the reaction, decreases overall. In addition, the reason the amount of CH production is lower than that of OPC is because CH is consumed during the reaction of SP.

In the case of LSP mixed specimens, the amount of AFt amount was similar to that of OPC and SP specimens at 1, 8H curing. However, in 1-day curing, LSP5 was 1%, and LSP10 was 2.8% higher than the OPC specimen and 2.5 to 4.5% higher than the SP specimens. In 7 days of curing, LSP5 was found to be about 0.5% larger, and LSP10 was about 1% larger than OPC and SP specimens. In 28 days of curing, all of the OPC, SP, and LSP specimens showed similar AFt product amounts. On the other hand, in the case of the AFm, it was confirmed that the LSP specimens were smaller than the OPC and SP specimens at the initial age (1, 7 days), while it was further increased at 28 days curing. Through this, it was confirmed that the incorporation of LSP generates a large amount of AFt at the initial age, and it was found that it did not significantly affect the generation of CH.

The AFt production of SP20LSP5 was slightly higher at the initial age (1 day, 7 days) than OPC but showed a lower tendency at the 28 days. It is inferred that when LSP was added, AFm in the form of a more stable monocarbonate type was generated, and thus monosulfate was reduced, which affected the AFt production. As a result, the addition of LSP is not effective for the formation of monocarbonate (AFm) and helps improve the initial reactivity of hydration by the nucleation effect. Furthermore, the stabilization of LSP and reaction with aluminate minerals improved the pore of the matrix [26].

**Figure 3:**
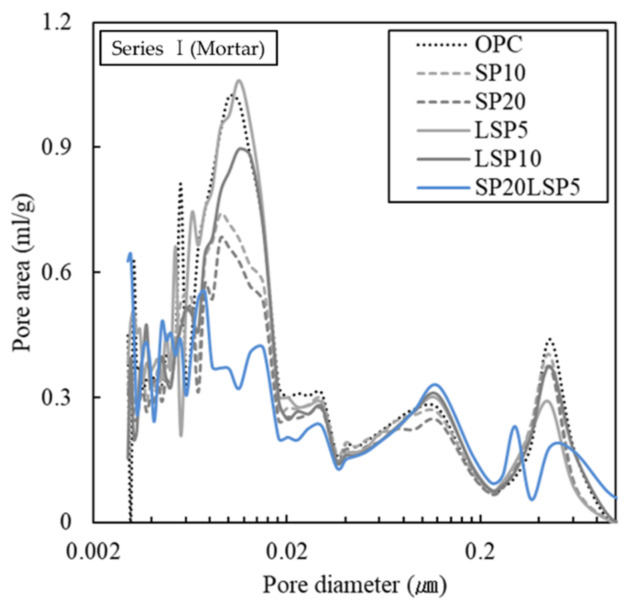
MIP measurement result of binder specimens at 28 days.

The MIP results of the shotcrete binder containing SP and LSP obtained after 28 days of curing are shown in Figure 3 and Table 5. The pore size is classified into large capillary pores (0.05–10 μm), medium capillary pores (0.01–0.05 μm), and Gel pores (0.01 μm or less) [27].

With increasing SP content, the capillary pores and gel pores were decreased, suggesting the formation of a denser matrix, which is possibly due to the latent hydraulic reaction of SP forming C-S-H. Furthermore, the particle size of SP is comparatively smaller than that of OPC, which owing to its filling effect reduced the capillary pores [28,29].

Upon addition of LSP, no clear difference in pore distribution of the LSP5 sample and OPC sample can be observed, but the LSP10 sample reduced gel pores and capillary voids by about 10%. The addition of LSP reacts with the aluminate phase in the OPC and forms carboaluminate, which thereby stabilizes the ettringite and reduces the gel pores as well as capillary pores because it also reacts with the aluminate mineral contained in the slag or pozzolan material to reduce the voids [30,31].

In the case of SP20LSP5, it was confirmed that capillary voids were significantly reduced compared to OPC. This is associated with the formation of C-S-H, filling effect due to the latent hydraulic property of SP, ettringite stabilization by LSP, and reaction with aluminate minerals [32].

#### 3.1.3. Analysis Result of Penetration Resistance and Compressive

**Figure 4:**
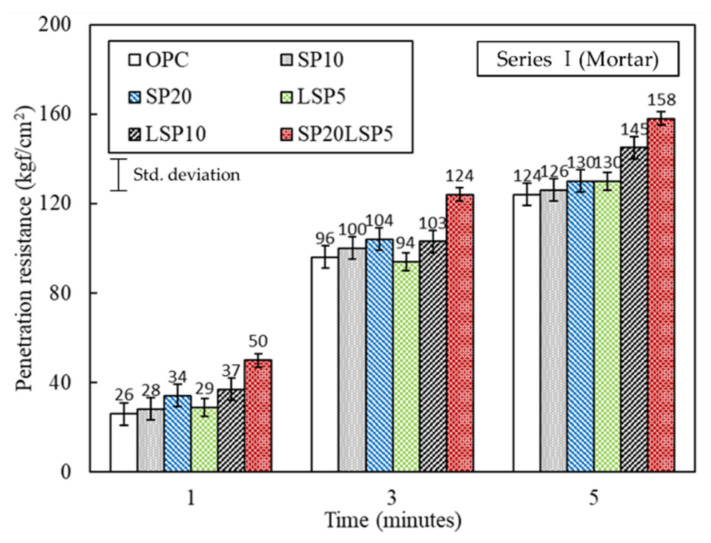
Penetration resistance value of mortar specimens.

The penetration resistance of mortar is shown in Figure 4. From the results, it is confirmed that the penetration resistance of SP substituted mortar is almost the same as that of OPC. Additionally, no significant effect on the setting of mortar containing up to 20% SP is observed. The penetration resistance of the LSP5 sample is not significantly different from that of the OPC. However, the LSP10 specimens showed a penetration resistance value that was 15 kgf/cm^2^ higher than OPC in 5 min. On the other hand, the SP20LSP5 specimen showed a penetration resistance value of about 25 to 30 kgf/cm^2^ higher than that of OPC at 1–5 min. Therefore, it was confirmed that the hardening time decreased when LSP and SP were mixed together. Therefore, it is considered that there will be a blending effect that affects the hardening time.

**Figure 5:**
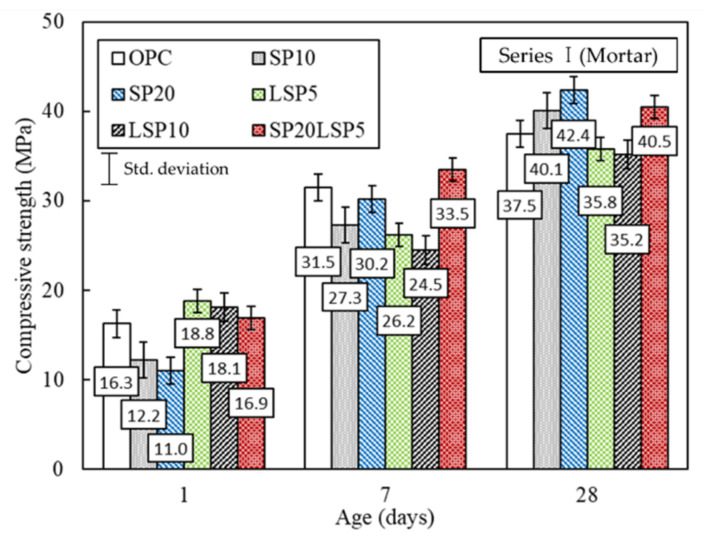
Compressive strength of mortar specimens at age.

As shown in Figure 5, the early strength of the SP-mixed test specimen was found to be about 20 to 30% lower than that of OPC, but 28 days strength increased than the strength of OPC. This is believed to be due to the Latent Hydraulic Activity of SP. The 1-day compressive strength of the specimens containing LSP was higher than OPC, but the compressive strength at the age of 7 and 28 days was observed to be lower than OPC. On the other hand, the SP20LSP5 specimen tended to be similar or higher than the strength of OPC specimen in all ages, and no decrease in strength due to mixing of the mixture was observed considering measurement errors.

**Figure 6:**
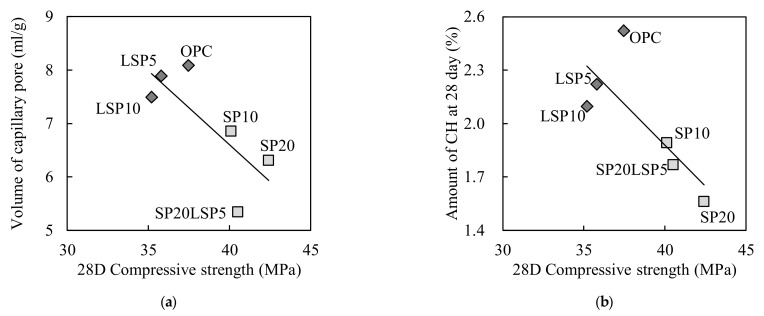
Relationship between compressive strength and binder type of mortar specimen at 28 day: (**a**) Volume of capillary pore; (**b**) Amount of CH.

In order to analyze the effect of SP and LSP on 28 days compressive strength, the CH production and volume of the capillary pore are shown in relation to the compressive strength in Figure 6. In the sample containing SP, both capillary voids and the amount of CH production were decreased. However, the 28-day compressive strength was improved despite the decrease in the amount of CH production. Although the capillary pores of samples containing LSP were lowered than that of OPC, the 28 days compressive strength was decreased. The decreased 28 days compressive strength could be associated with the reduction of the amount of C-S-H hydrate production with the decreased OPC in a matrix, reducing the adhesion between particles.

### 3.2. Hardened Properties and Durability of the Shotcrete

#### 3.2.1. Methods of Shotcrete Experiment

In order to perform the compressive strength test, the cylindrical specimens with a diameter of 100 mm and height of 200 mm were used, and the test was performed in accordance with ASTM C873 and ASTM C39, and an average value of three specimens was obtained [33,34]. The chloride diffusion coefficient of shotcrete was evaluated by NT-BUILD 492 [35]. Before executing the experiment, the central part of cylindrical specimens was cut to a thickness of 50 mm and pretreated in a Ca(OH)_2_ saturated solution for 24 h while the vacuum was maintained for 3 h. For the cut specimens, the anode is exposed to a 0.3 N NaOH aqueous solution (NaOH 12 g in 1 L of water), and the cathode is exposed to a 10% NaCl aqueous solution (NaCl 100 g + distilled water 900 g) to measure the initial current value when 30 V voltage is applied. The applied voltage level and time for testing were selected based on the measured current value. The chloride diffusion coefficient is calculated using Equations (3)–(5) after measuring the depth of discolored to silver gray when 0.1 M of AgNO_3_ solution was sprayed on the cut surface after cutting the samples.

(3) $$D_{nssm}=\frac{RT}{zFE}\times \frac{X_{d}-\alpha \sqrt{X_{d}}}{t}$$

(4) $$E=\frac{U-2}{L}$$

(5) $$\alpha =2\sqrt{\frac{RT}{zFE}}\times erf^{-1}\left(1-\frac{2C_{d}}{C_{0}}\right)$$

where *D_nssm_*: non-steady-state migration coefficient (m^2^/s); *R* is the gas constant, *R* = 8.314 J/(K·mol); *T* is the average value of the initial and final temperatures in the anolyte solution, *z* is the absolute value of the ion valence for chloride, *z* = 1; *F* is the Faraday constant, *F* = 9.648 × 10^4^ J/(V·mol); *L* is the thickness of the specimen m; *U* is the absolute value of the applied voltage, V, K; *X_d_* is the average value of the penetration depths, m; *t* is the test duration, seconds; *erf*^−1^ is the inverse of the error function; *C*_0_ is the chloride concentration in the catholyte solution, *C*_0_ ≈ 2 N; and *C_d_* is the chloride concentration at which the color changes, *C_d_* ≈ 0.07 N for OPC concrete.

The freeze–thaw resistance test of shotcrete was performed in accordance with ASTM C666/C666M [36]. Visual observation and relative dynamic modulus of the specimens subjected to water curing was performed every 30 cycles so that water could be saturated in the pores of all test specimens. The relative dynamic modulus was calculated by Equation (6).

(6) $$P_{c\;}=\left(\frac{n_{1}^{2}}{n^{2}}\right)\times 100$$

Here, *P_c_* is the relative dynamic modulus (%) after the freeze–thaw *C* cycle, *n* is the initial primary resonance frequency (Hz), and n_1_ is the primary resonance frequency (Hz) after the c-cycle in freeze–thaw.

#### 3.2.2. Results of Shotcrete Experiment

**Figure 7:**
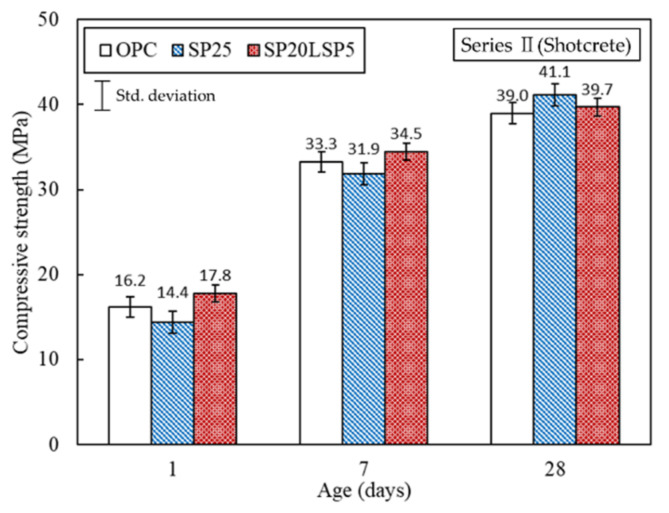
Compressive strength of shotcrete.

As shown in Figure 7, the SP20LSP5 specimen showed a compressive strength value similar to that of the OPC specimen at the initial age (1, 7 days) and a slightly higher value compared to the SP25 specimen. The sample SP25 showed slightly higher 28 days strength than OPC and SP20LSP5 samples. This can be associated with the hydration reaction mechanism of the mortar being reproduced, and the low reactivity of SP during the early reaction is compensated by the addition of LSP, and a denser microstructure is created by SP at the end of the reaction. As a result, it was confirmed that the simultaneous use of LSP and SP did not cause a decrease in the initial strength of the shotcrete but rather exhibited a strength behavior similar to that of OPC specimens.

**Figure 8:**
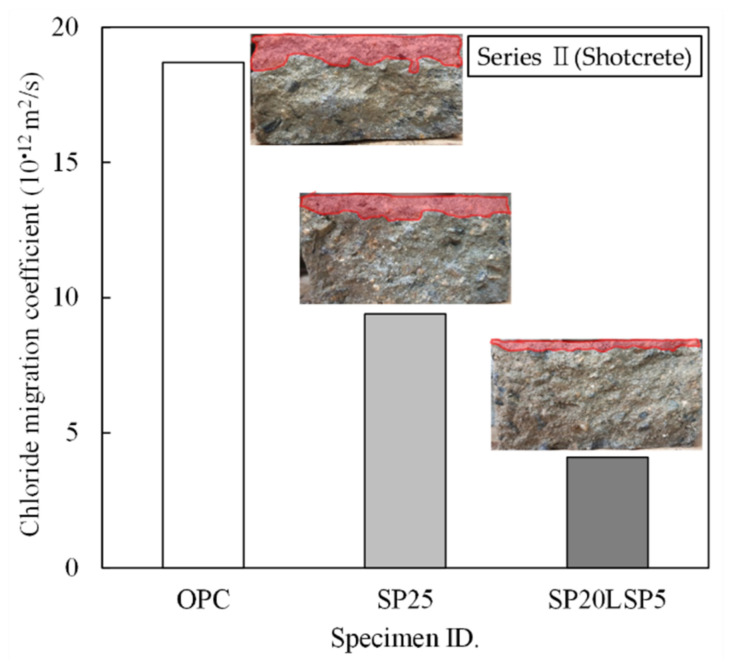
Chloride migration coefficient of shotcrete.

As shown in Figure 8, the chloride ion diffusion coefficient is high for OPC (18.7 × 10^−12^ m^2^/s), followed by SP (9.4 × 10^−12^ m^2^/s), and SP20LSP5 (18.7 × 10^−12^ m^2^/s). Chloride ion diffusion in SP is about lower 50% than OPC, SP20LSP5 is about 20% lower than OPC. This could be due to the structure being densified with the addition of fine powder in blast furnace slag, and at the same time, the aluminum oxide (Al_2_O_3_) component present in the blast furnace slag reacted with the chloride ion to form Friedel salts (3CaO·Al_2_O_3_·CaCl_2_·10H_2_O). In addition, the chloride ion diffusion coefficient of the SP20LSP5 specimen is considered to be due to the filling effect of fine limestone powder with high fineness [37,38,39].

Table 6 shows the surface scaling of specimens exposed to freezing-thawing. The surface deterioration of the OPC specimen increased significantly over the course of cycles, whereas the surface deterioration of the SP25 and SP20LSP5 specimens is comparatively less. Results suggested that LSP owing to its greater reactivity, not only serves as a filler but also improves the density of the matrix [18,19].

**Figure 9:**
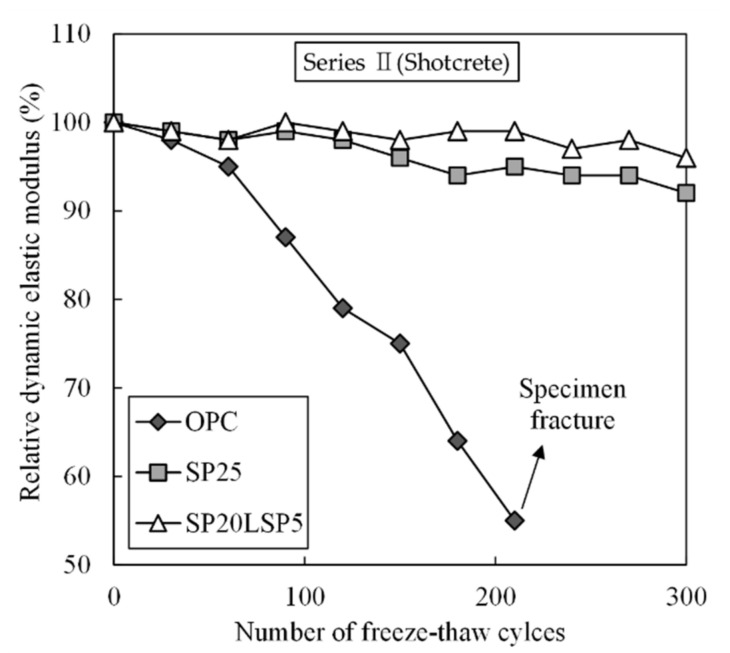
Freeze–thaw resistance performance of shotcrete.

Figure 9 shows the freeze–thaw resistance performance of shotcrete. The relative dynamic modulus of OPC decreased significantly after 60 cycles and decreased to less than 60% after 200 cycles. The SP and SP20LSP5 test specimens showed more than 90% of the relative dynamic modulus until 300 cycles without significant deterioration. Results show the samples incorporating SP and LSP improved the freeze–thaw resistance, which can be associated with the formation of dense internal structure than that of OPC [40,41].

## 4. Conclusions

Based on the experimental results, the following conclusion can be drawn:

1. Hydration analysis showed that the ettringite was preferentially produced by hydration reaction with CA (accelerator used for rapid setting) and the amount of CH produced was similar in all samples. In addition, LSP showed excellent performance at early ages, and when used with SP, the reactivity at the early stages of hydration was improved.
2. Incorporation of SP improves 28 days compressive strength caused by capillary pores and gel pores reduction. Partial substitution of LSP stabilized the ettringite and reduced the pores as reacted with aluminate minerals.
3. No delay in the setting of mortar was observed with the addition of SP addition, and the setting was increased as the LSP was added, increasing the penetration resistance. When the combination of SP and LSP were mixed, the setting time increased, increasing the penetration resistance and strength of shotcrete mortar.
4. Specimen containing SP and LSP together exhibits high chloride resistance and freeze–thaw resistance due to the decrease in capillary pores. The combined use of SP and LSP was found to be effective in improving the durability of shotcrete.

Through the present study, it was confirmed that when LSP and SP are simultaneously used as the CA accelerator for the shotcrete, an initial strength decrease is prevented due to a blending effect of the LSP and the SP and durability of the shotcrete is improved. However, it is judged that micro-structure analysis and synergy mechanism research through SEM is necessary to clearly identify the blending effects of LSP and SP through future research. Based on this, it is necessary to analyze field applicability, economy, and usability by actual field application experiments.

## Figures and Tables

**Table 1:** Chemical composition of materials.

Materials	Chemical Composition (%)							
	CaO	SiO_2_	Al_2_O_3_	Fe_2_O_3_	SO_3_	MgO	K_2_O	L.O.I ^e^
OPC ^a^	63.9	21.9	5.2	3.6	2.2	2.0	1.0	0.2
SP ^b^	37.4	34.1	14.9	1.4	3.2	6.8	0.6	1.6
LSP ^c^	43.7	10.9	3.7	1.8	0.7	1.3	1.0	36.9
SC ^d^	47.8	3.3	35.1	0.9	0.1	0.9	0.2	11.7

^a^ OPC: Ordinary Portland cement; ^b^ SP: Blast-furnace slag powder; ^c^ LSP: Limestone powder; ^d^ SC: Calcium aluminate accelerator; ^e^ L.O.I: Loss of ignition.

**Table 2:** Experimental plan.

Series	Specimen ID.	Type	Binder (wt.%)			Experimental Plan
			OPC	SP	LSP	
I	OPC	Mortar	100			- Hydrate analysis (XRD) - Pore distribution analysis (MIP) - Penetration resistance (1, 3, 5 min.) - Compressive strength (1, 7, 28 days)
	SP10		90	10		
	SP20		80	20		
	LSP5		95		5	
	LSP10		90		10	
	SP20LSP5		75	20	5	
II	OPC	shotcrete	100			- Compressive strength (1, 7, 28 days) - Chloride migration coefficient - Freeze–thaw resistance
	SP25		75	25		
	SP20LSP5		75	20	5	

**Table 3:** Mix proportion of Series I (Mortar experiment).

Series	W/B ^a^ (%)	B:S ^b^	Binder (g)	Water (g)	SC ^c^ (B × wt.%)
I (Mortar)	50	1:3	450	225	5.0

^a^ W/B = Water/binder ratio; ^b^ B/S = Binder/sand ratio, sand is ISO standard sand; ^c^ SC = Quick setting admixture for shotcrete (calcium aluminate accelerator).

**Table 4:** Mix proportion of Series II (Shotcrete experiment).

W/B	S/a ^a^ (%)	Unit Weight (N/m^3^)						Admixture (B × wt.%)	
		Water	Binder			Sand	Gravel	AD ^b^	SC ^c^
			OPC	SP	LSP				
0.43	60	190	441			1015	695	1.0	5.0
			331	110					
			331	88	22				

^a^ S/a: Sand/total aggregate ratio; ^b^ AD: Admixture; ^c^ SC: quick setting admixture for shotcrete (calcium aluminate accelerator).

**Table 5:** Volume classification by pore size of binder type at 28 days (Series I).

Classification of Pores		OPC	SP10	SP20	LSP5	LSP10	SP20 LSP5
Capillary pores	Large (0.05–10 μm)	2.39	2.34	2.15	2.23	2.36	2.32
	Medium (0.01–0.05 μm)	5.70	4.52	4.15	5.66	5.13	3.02
Gel pores (≤0.01 μm)		9.79	8.79	7.93	10.6	8.76	8.91
Total pores		17.9	15.6	14.2	18.5	16.3	14.3

**Table 6:** Surface scaling of shotcrete specimens exposed to freezing-thawing.

OPC				SP25				SP20LSP5			
0 cycle	120 cycles	210 cycles	300 cycles	0 cycle	120 cycles	210 cycles	300 cycles	0 cycle	120 cycles	210 cycles	300 cycles
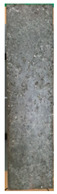	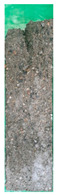	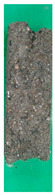	-	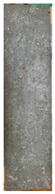	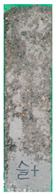	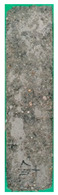	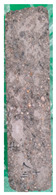	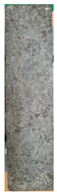	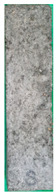	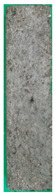	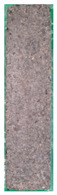

## References

[1] Thomas A. (2012). Sprayed Concrete Lined Tunnel.

[2] American Concrete Institute (1990). Specification for Materials, Proportioning, and Application of Shotcrete, ACI Committee 506, ACI 506.2-77.

[3] Hemphill G.B. (2012). Practical Tunnel Construction.

[4] Maltese C., Pistolesi C., Bravo A., Cella F., Cerulli T., Salvioni D. (2007). A case history: Effect of moisture on the setting behaviour of a Portland cement reacting with an alkali-free accelerator. Cem. Concr. Res..

[5] Liu X., Zhu Y., Li X., Du G.P., Song Z.P. (2009). Experimental research on single-layer tunnel of steel fiber shotcrete. Rock Soil Mech..

[6] Ghosh S.N. (1991). Cement and Concrete Science & Technology.

[7] Kurdowski W. (2014). Cement and Concrete Chemistry.

[8] Lea F.M. (1971). The Chemistry of Cement and Concrete.

[9] Leung C.K.Y., Lai R., Lee A.Y.F. (2005). Properties of wet-mixed fiber reinforced shotcrete and fiber reinforced concrete with similar composition. Cem. Concr. Res..

[10] Kim H., Moon H., Kim J.-H., Chung C.-W. (2014). Influences of Slag Replacement on the Properties of Shotcrete Using a Slurry-Type Set Accelerator. J. Korea Inst. Build. Constr..

[11] Edmonds R.N., Majumdar A.J. (1989). The hydration of mixtures of monocalcium aluminate and blastfurnace slag. Cem. Concr. Res..

[12] Gosselin C. (2009). Microstructural Development of Calcium Aluminate Cement Based Systems with and without Supplementary Cementitious Materials (No. THESIS).

[13] Wang D., Shi C., Farzadnia N., Shi Z., Jia H. (2018). A review on effects of limestone powder on the properties of concrete. Constr. Build. Mater..

[14] Schmidt M., Harr K., Boeing R. (1993). Blended cement according to ENV 197 and experiences in Germany. Cem. Concr. Aggreg..

[15] Moir G.K., Kelham S. (1999). Developments in the manufacture and use of Portland limestone cement. Spec. Publ..

[16] Cussino L., Negro A. (1980). Hydration of aluminous cement in the presence of silicic and calcareous aggregates. International Congress on the Chemistry of Cement.

[17] Fentiman C.H. (1985). Hydration of carbo-aluminous cement at different temperatures. Cem. Concr. Res..

[18] Juradin S., Vlajić D. (2015). Influence of cement type and mineral additions, silica fume and metakaolin, on the properties of fresh and hardened self-compacting concrete. Adv. Struct. Mater..

[19] Uysal M., Sumer M. (2011). Performance of self-compacting concrete containing different mineral admixtures. Constr. Build. Mater..

[20] (2017). Standard Specification for Standard Sand.

[21] (2016). Standard Specification for Materials for Shotcrete.

[22] (2018). Standard Test. Method for Determination of the Proportion of Phases in Portland Cement and Portland-Cement Clinker Using X-ray Powder Diffraction Analysis.

[23] (2018). Standard Test. Method for Determination of Pore Volume and Pore Volume Distribution of Soil and Rock by Mercury Intrusion Porosimetry.

[24] (2016). Standard Test Method for Time of Setting of Concrete Mixtures by Penetration Resistance.

[25] (2017). Standard Test Method for Compressive Strength of Hydraulic Cement Mortars.

[26] Balonis M., Glasser F.P. (2009). The density of cement phases. Cem. Concr. Res..

[27] Mindess S., Young J.F., Darwin D. (2003). Concrete.

[28] Rashad A.M., Sadek D.M. (2016). An investigation on Portland cement replaced by high-volume GGBS pastes modified with micro-sized metakaolin subjected to elevated temperatures. Int. J. Sustain. Built Environ..

[29] Özbay E., Erdemir M., Durmuş H.İ. (2016). Utilization and efficiency of ground granulated blast furnace slag on concrete properties—A review. Constr. Build. Mater..

[30] De Weerdt K., Kjellsen K.O., Sellevold E., Justnes H. (2011). Synergy between fly ash and limestone powder in ternary cements. Cem. Concr. Compos..

[31] Antoni M., Rossen J., Martirena F., Scrivener K. (2012). Cement substitution by a combination of metakaolin and limestone. Cem. Concr. Res..

[32] Soroka I., Setter N. (1977). The effect of fillers on strength of cement mortars. Cem. Concr. Res..

[33] (2015). Standard Test Method for Compressive Strength of Concrete Cylinders Cast in Place in Cylindrical Molds.

[34] (2018). Standard Test Method for Compressive Strength of Cylindrical Concrete Specimens.

[35] (1999). Concrete, Mortar and Cement-Based Repair Materials: Chloride Migration Coefficient from Non-Steady-State Migration Experiments.

[36] (2015). Standard Test Method for Resistance of Concrete to Rapid Freezing and Thawing.

[37] Lee S.T., Kim S.S., Kim D.G., Park K.P. (2013). Effect of Types of Accelerators and Replacement Levels of GGBFS on the Performance of Shotcrete Mortars. J. Korea Inst. Struct. Maint. Insp..

[38] Sun J., Chen Z. (2018). Influences of limestone powder on the resistance of concretes to the chloride ion penetration and sulfate attack. Powder Technol..

[39] Panesar D.K., Zhang R. (2020). Performance comparison of cement replacing materials in concrete: Limestone fillers and supplementary cementing materials—A review. Constr. Build. Mater..

[40] Deja J. (2003). Freezing and de-icing salt resistance of blast furnace slag concretes. Cem. Concr. Compos..

[41] Stark J., Ludwig H.M. (1997). Freeze-thaw and freeze-deicing salt resistance of concretes containing cement rich in granulated blast furnace slag. ACI Mater. J..

